# Recent progress in chronic pain-related negative emotions and cognitive dysfunction: insights into the mechanisms underlying neural circuitry

**DOI:** 10.3389/fncel.2026.1759181

**Published:** 2026-02-05

**Authors:** Feng-Xian Hu, Hong-Yun Wu, Yu Wang, Yun-ling Zhang, Wen-Qiang Cui

**Affiliations:** 1 First College of Clinical Medicine, Shandong University of Traditional Chinese Medicine, Jinan, China; 2 Department of Neurology, Affiliated Hospital of Shandong University of Traditional Chinese Medicine, Jinan, China; 3 Xiyuan Hospital of China Academy of Chinese Medical Sciences, Beijing, China

**Keywords:** pain, comorbidity, cognitive dysfunction, negative emotions, brain nuclei, neural circuits

## Abstract

Pain is a complex sensory and affective experience that is frequently accompanied by comorbid conditions such as anxiety, depression, fear, and cognitive dysfunction, collectively exacerbating patient suffering and disease burden. Despite significant advancements in pain research, the mechanisms underlying chronic pain and its related negative emotions remain inadequately understood. The cerebral cortex, lateral habenula (LHb), thalamus, amygdala, parabrachial nucleus (PBN), hippocampus, and locus coeruleus (LC) are widely associated with chronic pain, chronic pain-related negative emotions, and cognitive dysfunction. In this review, we summarize recent research on the functions of various brain nuclei and their subregions in chronic pain and related negative emotions and cognitive dysfunction from the perspective of neural circuits. By delineating these circuit-level mechanisms, we aim to provide insights that may inform the development of more effective strategies for the clinical diagnosis and treatment of chronic pain and comorbid emotional and cognitive dysfunctions.

## Graphical abstract

**Figure:**
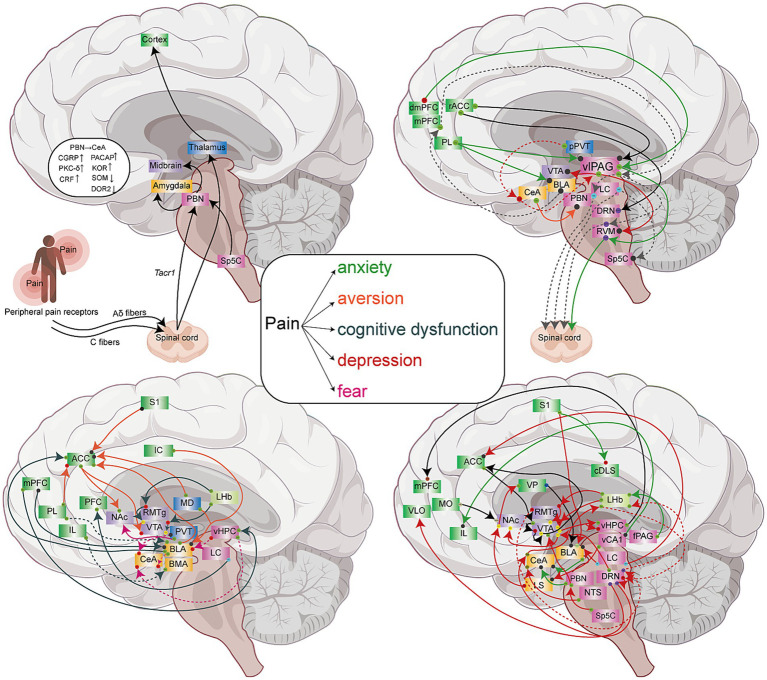
Chronic pain (pathological neuropathic pain models and chronic inflammatory pain models) induces the generation and development of anxiety, depression, fear, aversion, and cognitive impairments by activating/inhibiting specific neural circuits in the brain.

## 1 Introduction

Pain is a distressing sensory and emotional experience that may be caused by actual or potential harm to tissues (Raja et al., 2020). It is multidimensional, including sensory-discriminative, affective-motivational, and cognitive-evaluative components. Chronic pain affects approximately 30% of people (Cohen et al., 2021), and is commonly associated with anxiety, depression, fear, and cognitive dysfunction, which severely impair the mental and physical health of individuals (Aaron et al., 2025; Shen et al., 2023; Stegemann et al., 2023; Usdin and Dimitrov, 2016; Bushnell et al., 2013; Yao et al., 2023). Consequently, a comprehensive understanding of the psychological and neural mechanisms underlying chronic pain is crucial for the development of effective intervention strategies and the enhancement of patients’ quality of life.

Various brain nuclei have demonstrated critical functions in chronic pain-related negative emotions and cognitive dysfunction, which include the cerebral cortex (Usdin and Dimitrov, 2016), habenula (LHb) (Cui et al., 2020; Hu et al., 2020), thalamus (Liang H. Y. et al., 2020; Liang S. et al., 2020), hypothalamus (Li et al., 2023), amygdala (Jiang et al., 2021; Li and Sheets, 2020; Mazzitelli et al., 2022; Usdin and Dimitrov, 2016), parabrachial nucleus (PBN) (Zhang et al., 2021; Zhou et al., 2021), hippocampus (HPC) (Kami et al., 2022; Lv et al., 2024) and locus coeruleus (LC) (Suárez-Pereira et al., 2022; Usdin and Dimitrov, 2016). Chronic pain interferes with normal brain functions across various regions, resulting in adverse emotional states and cognitive impairments, especially in cases of neuropathic and inflammatory pain (Liu D. et al., 2025; Liu Y. et al., 2025; Zhao T. et al., 2025; Zhou et al., 2021). The direct or indirect connectivity between these brain nuclei and the extensive functional recombination of different brain regions produced by pain may be one of the causes of chronic pain-related negative emotions and cognitive dysfunction (Kisler et al., 2016; McIlwrath et al., 2020; Raja et al., 2020). Despite extensive research on the neural circuits involved, the intricate nature of these mechanisms, coupled with current methodological limitations, impedes a comprehensive understanding of how integrated neural networks coordinate to mediate chronic pain and its associated emotional symptoms and cognitive dysfunction. Given the intricate neurobiological mechanisms underlying this interaction, elucidating the neural substrates of pain-induced negative emotions represents a critical challenge in both basic research and clinical practice.

This review consolidates findings from animal studies to provide an extensive overview of the neural circuit mechanisms underlying emotional abnormalities and cognitive impairments associated with chronic pain, with a particular focus on recent advancements in the field. Utilizing the conceptual framework of “upward transmission → central integration → downward regulation,” it delineates the comprehensive pathway of pain signals from peripheral origins to their processing in emotional and cognitive domains, thereby offering a systematic perspective for understanding the mechanisms of pain comorbidity. It aims to bridge the gap between established macro-level concepts and the emerging micro-level understanding, guiding future research toward circuit-based therapeutics and biomarkers.

## 2 Primary processing of pain information

### 2.1 Spinal cord

In the context of sustained injury, the components of the peripheral and central nervous systems involved in the pain transmission pathway demonstrate significant plasticity, which amplifies pain signals and may induce hypersensitivity reactions. Should these plastic changes remain, they have the potential to result in chronic pain (Basbaum et al., 2009). In the peripheral nervous system, sensory information is predominantly conveyed through three types of fibers: Aβ, Aδ, and C fibers. Of these, Aδ and C fibers are collectively identified as nociceptive receptors, commonly referred to as “pain fibers,” due to their capacity to respond to noxious mechanical, thermal, or chemical stimuli. Notably, C fibers, characterized as the smallest and unmyelinated primary afferent neurons, exhibit the slowest conduction velocity and the highest activation threshold among the three fiber types. This distinct property allows them to selectively respond to intense nociceptive stimuli and facilitate the transmission of persistent pain signals (Chen and Tang, 2024; D’Mello and Dickenson, 2008).

**Figure 1:**
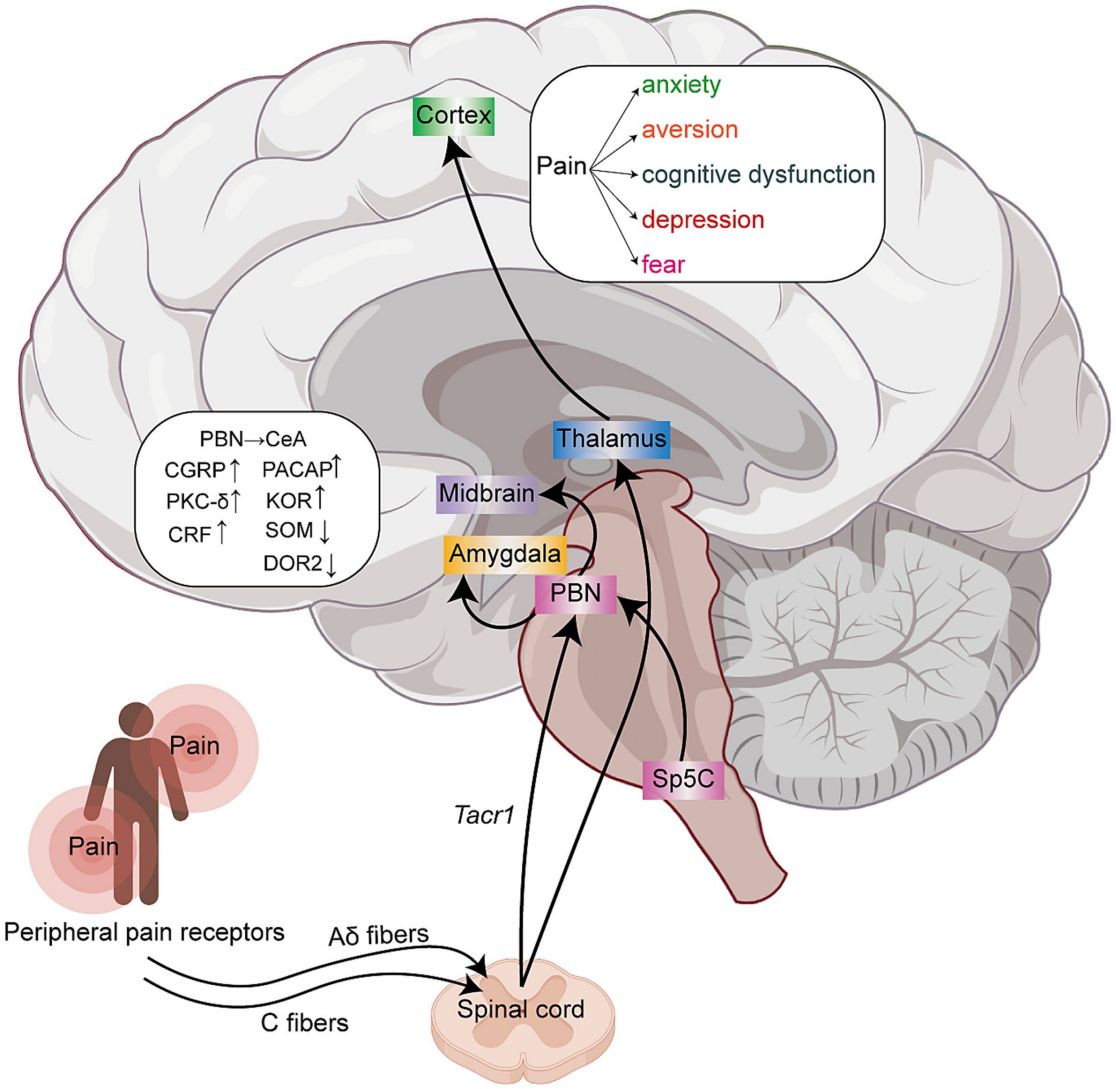
Schematic diagram of the ascending pain signal transmission pathways. Peripheral nociceptors receive pain information, which is primarily transmitted via Aδ and C fibers to the spinal cord. The signals are then relayed from the spinal cord to higher brain centers. Pain information ascends through multiple neural pathways, including the spinal cord → parabrachial nucleus → amygdala pathway, the spinal cord → parabrachial nucleus → midbrain pathway, the spinal cord → thalamus → cortex pathway, and the Sp5C → parabrachial nucleus → midbrain (VTA) circuit. These pathways play a key role in converting nociceptive signals into emotional responses, thereby promoting the occurrence and persistence of pain-related maladaptive behaviors.

The spinal cord (SC) serves as the initial relay station for conveying sensory information from the peripheral nervous system to the brain. Under continuous injury stimulation, adaptive dysplastic changes in synapses and abnormal neuronal activities can occur within the spinal cord. The latest research has revealed the spatial changes in genes, cell groups, intracellular molecular networks, cell-to-cell interactions, and cell-intracellular connections within the SC during neuropathic pain, which are closely related to the regulation of pain (Dong et al., 2025). The spinal projection neurons can further transmit the pain information to higher brain regions (D’Mello and Dickenson, 2008; Dong et al., 2025) (Figure 1).

Among these regions, the PBN is a key downstream target, forming a critical spinal → parabrachial → thalamic circuit that relays injury-related signals to supraspinal regions. Studies have found that *tachykinin receptor 1* (*Tacr1*) is an important marker of SC projection neurons, which project and activate PBN*^Tacr1^* neurons under pain stimulation (Barik et al., 2021; Deng et al., 2020). Chemical activation of PBN*^Tacr1^* neurons induces mechanical pain in mice and makes them irritable, anxious and “hyper-vigilant” (Barik et al., 2021). Studies have also revealed a spinal → parabrachial → midbrain circuit that modulates dopaminergic (DAergic) neurons, providing insights into how chronic pain influences learning and motivated behaviors (Yang et al., 2021). Tsou et al. (2023) demonstrated that PBN inputs also regulate non-DAergic ventral tegmental area (VTA) populations, such as PBN-mediated activation of VTA glutamatergic neurons, which possibly mediating the regulation of negative emotions (such as fear, stress, and disgust).

*Tacr1* neurons can further transmit nociceptive information to the thalamus and hypothalamus, including intralaminar thalamic nuclei, midline thalamus, lateral hypothalamic area (LHA), and parasubthalamic nucleus (Barik et al., 2021; Deng et al., 2020). Nociceptive signals are transmitted via thalamocortical projections, distributing pain information to multiple cortical regions, including the primary and secondary somatosensory cortices, insula, anterior cingulate cortex (ACC), and prefrontal cortex (PFC) (D’Mello and Dickenson, 2008; Tracey and Mantyh, 2007). These areas collectively form the “pain matrix,” which encodes sensory-discriminative, affective-motivational, and cognitive-evaluative dimensions of pain (D’Mello and Dickenson, 2008; Tracey and Mantyh, 2007). Given that many of these regions are also involved in emotion regulation and cognitive processing, their dysregulation under chronic pain conditions may contribute to the development of negative emotional states. This multi-level integration of nociceptive signals highlights the complex interplay between pain circuits and emotional circuits.

### 2.2 The spinal trigeminal subnucleus caudalis

The spinal trigeminal subnucleus caudalis (Sp5C), a crucial sensory processing center in the medulla, serves as the primary relay station for nociceptive information from the face and head (Kim H. K. et al., 2024). This region demonstrates significant neuronal activation, as evidenced by robust c-Fos expression, in both migraine and trigeminal pain models (Wu et al., 2022; Xiao et al., 2024). Zhang et al. (2021) identified a complete neural circuit, the Sp5C → lateral PBN (LPBN) → VTA pathway, that links craniofacial pain to depressive-like behaviors (Figure 1). Their study demonstrated that trigeminal neuralgia signals originate in the Sp5C, which activates glutamatergic projections from the LPBN to the VTA. This pathway causes excessive activation of VTA dopamine neurons, which directly drives pain-related negative emotions. These results establish Sp5C as a critical gateway transmitting pain signals to the brain’s emotion centers and highlight its potential as a therapeutic target for the emotional comorbidities of chronic pain.

## 3 The integration of pain sensation and emotional information

### 3.1 PBN is the key to the transmission of nociceptive information

The PBN has traditionally been considered a pivotal relay station for sensory information, situated in the dorsal upper region of the pons and encircling the lateral portion of the superior cerebellar peduncle (Chiang et al., 2019). The PBN is involved in transforming acute nerve injury into chronic pain (Li et al., 2025; Palmiter, 2024). It serves as the primary site for receiving direct nociceptive inputs from the spinal cord and is instrumental in the rapid and selective transmission of pain signals to limbic system structures, including the amygdala, hypothalamus, and the intralaminar nuclei of the thalamus (Deng et al., 2020; Gauriau and Bernard, 2002; Ke et al., 2024; Nakaya et al., 2025). In particular, the LPBN serves as a critical gateway for exteroceptive sensory information and represents the first site of integration for many affective and behavioral states (Goldstein et al., 2025). Glutamatergic neurons in the LPBN (LPBN^Glu^) are involved in basic pain transmission and neuropathic pain processing. Activating LPBN^Glu^ neurons or inhibiting local gamma-aminobutyric acid (GABAergic) interneurons causes significant hyperalgesia and aversive behaviors. In contrast, silencing glutamatergic activity or boosting GABAergic signaling reduces sensory hypersensitivity and emotional distress linked to neuropathic pain (Sun et al., 2020).

Of particular importance is the PBN’s projection to the central amygdala (CeA), which serves as a crucial interface for integrating somatosensory and affective signals. Nociceptive stimuli activate distinct PBN → CeA circuits that mediate both reflexive defensive responses and spontaneous affective-motivational reactions to pain (Torres-Rodriguez et al., 2023). At the molecular level, this process involves specialized calcitonin gene-related peptide-expressing neurons within the PBN that selectively transmit nociceptive information to the CeA, potentially encoding the affective valence of painful experiences (Han et al., 2015). Chronic neuropathic pain upregulates pituitary adenylate cyclase-activating polypeptide (PACAP) expression across the spino → parabrachio → amygdaloid pathway (Missig et al., 2017). Activation of PACAP neuronal projections from LPBN to CeA increases anxiety-like behaviors and mechanical pain sensitivity (Seiglie et al., 2023). These findings collectively position the PBN → CeA circuit as a promising therapeutic target for addressing both the sensory and affective components of chronic pain syndromes.

The CeA contains diverse GABAergic neuron subtypes expressing various molecular markers. Research has found that the projection of PBN to CeA is neuron-specific (Li and Sheets, 2020). PBN modulates somatostatin (SOM) and corticotropin-releasing hormone (CRH) neurons in CeA differentially by feeding monosynaptic excitatory to laterocapsular (CeLC), lateral (CeL), and medial (CeM) (Li and Sheets, 2020). Under nerve injury conditions, PBN promotes chronic pain-related responses by inhibiting CeA^SOM^ neurons and activating protein kinase C-delta (CeA^PKC-δ^) neurons (Wilson et al., 2019). Optogenetic activation of PKC-δ^+^ neurons is sufficient to induce mechanical hyperalgesia without eliciting anxiety-like behaviors in young mice. In contrast, optogenetic inhibition of SOM^+^ neurons similarly leads to mechanical hyperalgesia, whereas activation of SOM^+^ neurons triggers anxiety-like behaviors within the same cohort of animals (Chen et al., 2022; Chou et al., 2022).

Opioids exert analgesic effects and reduce negative emotional effects by inhibiting glutamatergic release at the PBN → CeLC synapse to reduce nociceptive information transmission (Kissiwaa et al., 2020). In a chronic pain model induced by complete Freund’s adjuvant (CFA), the inhibitory effect of delta opioid receptor 2 (DOR2) on the PBN → CeA pathway is lost after 21 days, resulting in persistent pain and anxiety-like behaviors in mice (Zhou et al., 2021). Electrophysiological studies reveal that kappa opioid receptor (KOR) activation in the CeA promotes aversive behaviors through a disinhibitory microcircuit. Optogenetic stimulation of parabrachial afferents to CeA corticotropin-releasing factor (CRF) neurons evokes a biphasic response: a direct monosynaptic excitatory postsynaptic current (EPSC) followed by a polysynaptic inhibitory postsynaptic current (IPSC) via feedforward inhibition. Critically, the KOR agonist U-69,593 selectively suppresses the polysynaptic IPSC without affecting the direct EPSC. This finding demonstrates that KOR activation specifically ablates feedforward inhibition, thereby disinhibiting CeA^CRF^ neurons to drive chronic pain-related aversive behaviors (Hein et al., 2021). Collectively, these findings demonstrate that the endogenous opioid system fine-tunes both sensory and affective aspects of pain through distinct receptors and circuit mechanisms. Targeted activation of DOR or antagonism of KOR in the CeA may thus represent a promising therapeutic strategy for chronic pain comorbid with negative emotional states.

### 3.2 Cortical remodeling of chronic pain

The transmission of information from sensory cortices to higher-order regions in the neocortex is essential for sensory processing, as these regions facilitate affective and cognitive responses. This integration of sensory and affective information is especially critical in the context of pain perception (Basbaum et al., 2009). In the initial phase of pain chronification, the somatosensory cortex (SSC), a key node for nociceptive information processing, undergoes crucial neuroplastic changes (Rainville et al., 2001; Singh et al., 2020; Takeda et al., 2022). The SSC is not only responsible for receiving and localizing noxious stimuli, but its neurons also undergo sensitization and reorganization under persistent pain input (Basbaum et al., 2009; Rainville et al., 2001; Valyear et al., 2020; Wang et al., 2025; Yi et al., 2025). This leads to an expansion of the pain representation area and heightened responses within the SSC, which is considered a fundamental peripheral and cortical mechanism underlying the transition from acute to chronic pain (Basbaum et al., 2009; Jenkins et al., 2022; Wang et al., 2025). As the pain state persists, these aberrant sensory signals are relayed to higher-order brain regions, notably the medial prefrontal cortex (mPFC), which is involved in advanced cognitive and emotional processing. Chronic inflammatory pain induces structural reorganization characterized by elevated perineuronal net density in both the SSC and mPFC (Mascio et al., 2022). Temporal dynamics reveal hypoactive SSC neurons during early injury phases transitioning to mPFC hyperexcitability in later stages, reflecting the progressive transformation from sensory processing to pain affective components (Zhang et al., 2022). Additionally, Kim H. K. et al. (2024) and Kim H. R. et al. (2024) showed an overall PFC deactivation due to hyper-negative GABAergic current in neuropathic pain, which strongly supports the PFC deactivation scenario. In summary, chronic pain-related cortical deactivation—driven by a shift in synaptic excitation/inhibition balance—underlies the expression of maladaptive pain-related behaviors.

As the principal cortical area responsible for the localization and discrimination of pain, the primary somatosensory cortex (S1) is integral to the encoding and integration of nociceptive information (Bushnell et al., 1999). In chronic pain states, there is an augmentation of functional connectivity between S1 and the ACC, with enhanced projections from S1 to ACC resulting in increased firing rates within the ACC. This facilitates the integration of sensory and affective components of pain (Singh et al., 2020). Importantly, pain conditions induce hyperexcitability of S1 hindlimb region glutamatergic neurons, whose specific projections to basolateral amygdala (BLA) cholecystokinin neurons have been shown to modulate depressive-like behaviors associated with chronic pain (Chen et al., 2025). Recent investigations at the circuit level have identified the glutamatergic pathway from the S1 to the GABAergic neurons in the caudal dorsolateral striatum (cDLS^GABA^) as a crucial neural substrate underlying the comorbidity of pain and anxiety in models using CFA (Jin et al., 2020). The excitatory circuit between S1^Glu^ and cDLS^GABA^ not only facilitates the maintenance of chronic pain but also induces anxiety-like behaviors, thereby providing a mechanistic explanation for the frequent co-occurrence of these conditions in clinical populations.

These findings underscore the dual role of the SSC, especially S1, in sensory processing and emotional modulation. The identified circuit mechanisms reveal how S1 serves as both a discriminative pain processor and an affective modulator through its ascending projections to limbic centers.

## 4 Cognitive dysfunction related to pain

**Figure 2:**
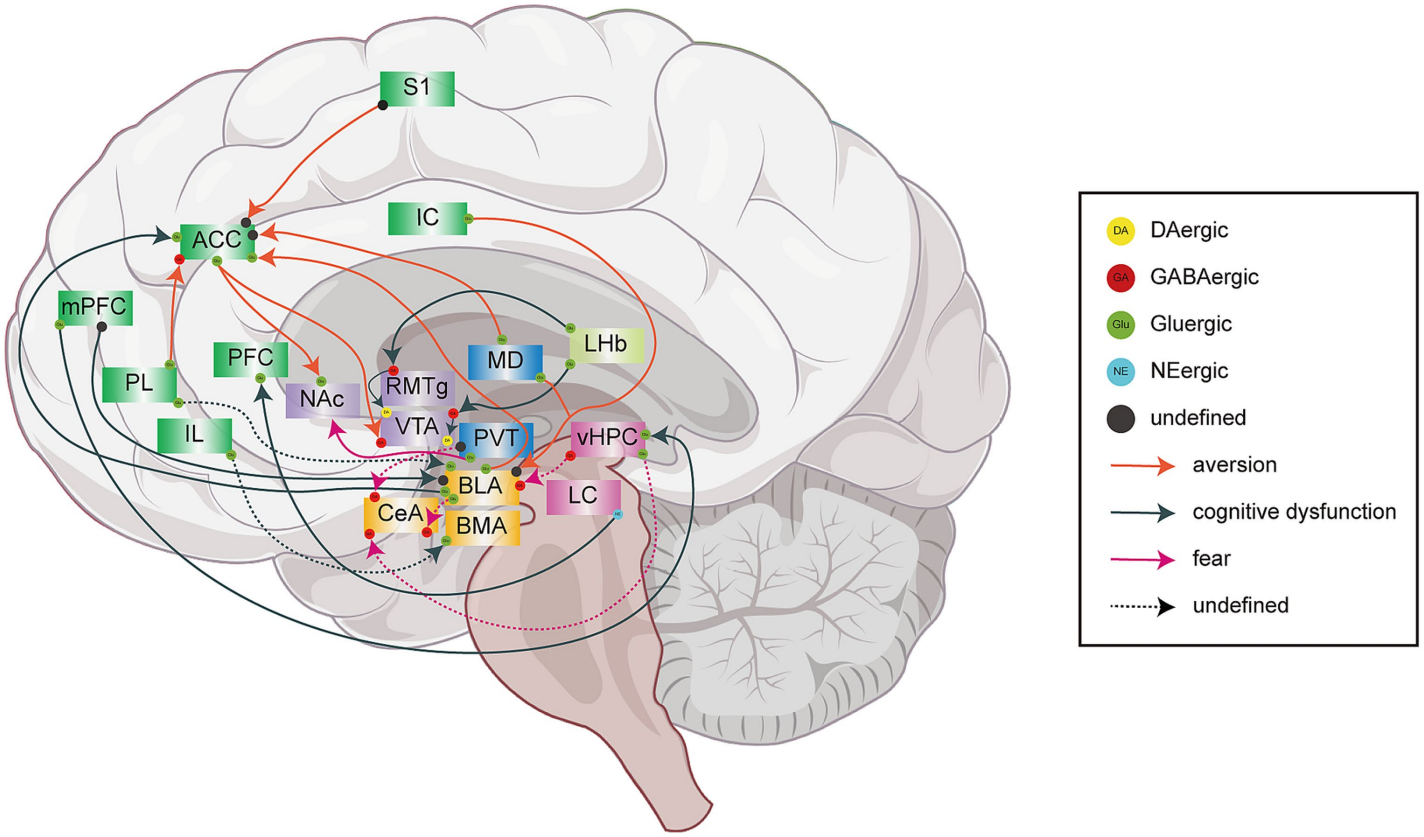
A schematic representation of neural circuits associated with behaviors related to chronic pain, including disgust, cognitive dysfunction, and fear. Solid lines indicate established neural circuits, while dashed lines represent neural circuits that may be involved in pain comorbid with emotional or cognitive impairments. Orange arrows denote neural circuits involved in the regulation of chronic pain-related aversion: PL → ACC, S1 → ACC, MD → ACC, BLA → ACC, ACC → NAc, ACC → VTA, MD/IC → BLA. Dark green arrows denote neural circuits involved in the regulation of chronic pain-related cognitive impairment: BLA → ACC, mPFC → BLA, mPFC → vHPC, LC → PFC, LHb → RMTg → VTA, LHb → VTA, etc. Rose-red arrows denote neural circuits involved in the encoding of chronic pain-related fear behaviors: PVT → CeA, BLA → CeA, vHPC → CeA, vHPC → BLA, etc.

Pain often leads to a decline in cognitive abilities, whereas cognitive processes like memory and attention influence chronic pain (Bushnell et al., 2013). Neural pathways involving both cortical and subcortical areas are essential in this interaction (Hasan et al., 2023; Liu D. et al., 2025; Liu Y. et al., 2025; Suto et al., 2014). In this review, we discussed the neural circuit mechanisms that underlie cognitive impairment induced by chronic pain (Figure 2).

### 4.1 mPFC

Chronic pain resulting from peripheral nerve injury is frequently comorbid with significant cognitive impairment mediated by PFC dysfunction (Shiers et al., 2018; Suto et al., 2014). This clinical manifestation is underpinned by profound neuroplastic reorganization within PFC circuits during the transition to chronic pain states (Shiers et al., 2018; Suto et al., 2014). Increased noradrenaline (NE) concentrations in PFC as a result of peripheral nerve damage cause PFC dysfunction and reduced cognitive function (Suto et al., 2014). This pathological phenomenon primarily originates from noradrenergic dysregulation caused by aberrant LC → PFC projections in neurogenic pain conditions. The connectivity between mPFC and BLA regulates pain-related cognitive dysfunction (Usdin and Dimitrov, 2016). This may be explained by the decreased activity of glutamatergic neurons in the prelimbic (PL) medial prefrontal cortex → BLA circuit, which interrupts memory consolidation. The enhanced activity of glutamatergic neurons in the infralimbic (IL) medial prefrontal cortex → BLA amygdala circuit promotes memory regression (Sun et al., 2023).

The HPC serves as a crucial neural hub that orchestrates multiple cognitive functions through its sophisticated circuitry and synaptic plasticity mechanisms (Karat et al., 2024; Liu Y. et al., 2024). Research has demonstrated that chronic constriction injury (CCI) leads to enduring pain and cognitive dysfunction. The PL glutamatergic neurons expressing calcium/calmodulin-dependent protein kinase-II α (CaMKIIα) play a selective and critical role in mPFC → hippocampal connectivity, which governs pain-related memory impairments (Cardoso-Cruz et al., 2019). Theta oscillation desynchrony between HPC and mPFC has been associated with cognitive dysfunction, increased synchrony is related to anxiety, whereas disrupted synchrony may indicate depression (Ruggiero et al., 2021; Soltani Zangbar et al., 2020). Nevertheless, additional research is necessary to thoroughly understand the neurobiological mechanisms underlying these relationships. The disruption of the neural network connection between HPC and mPFC and the abnormal rhythm between them can lead to the occurrence of pain-related negative emotions; however, the changes in the microcircuits and particular neurons involved remain to be determined.

### 4.2 LHb

CFA-induced inflammation and pain in rats led to reduced working memory performance, an increased omission percentage, and longer response latency; thus, suggesting cognitive dysfunction in CFA rats (Alemi et al., 2023). Peripheral nerve injury modifies the intra-LHb network activity associated with reward information processing and facilitates pain-related spatial working memory impairments by inducing heightened excitability of CaMKIIα neurons within the LHb (Cardoso-Cruz et al., 2024). The hyperactivity of the LHb results in dysfunction due to impaired signal transduction in VTA^DA^ circuits. The latest research has found that rostromedial tegmental (RMTg) is an important relay between LHb and VTA. Pain induces pain-related cognitive dysfunction and depression-like behaviors by activating the LHb^Glu^ → RMTg^GABA^ → VTA^DA^ neural circuit and altering VTA^DA^ activity (Liu D. et al., 2025; Liu Y. et al., 2025). Inhibition of the glutamatergic activity of LHb and its projection to VTA could reverse the difference between CFA and control rats (Alemi et al., 2023). Similarly, the LHb → VTA circuit regulates pain and associated cognitive dysfunction via the LHb^Glu^ → VTA^GABA^ → VTA^DA^ (Alemi et al., 2023). These data indicate that LHb regulates pain-related cognitive dysfunction by indirectly (RMTg^GABA^, VTA^GABA^) regulating the VTA-DA system, and the molecular mechanisms underlying the LHb → VTA circuit in pain-related cognitive dysfunction requires further investigation.

## 5 Pain aversion coding and fear avoidance

Pain aversion epitomizes a core dimension of suffering in chronic pain (Mercer Lindsay et al., 2021). The convergence of nociception and associated negative affect forms an aversive learning continuum that promotes survival through threat avoidance (Baliki and Apkarian, 2015). Evidence implicates the cortex and amygdala in mediating these adaptive neurobehavioral processes (Ji et al., 2018; Singh et al., 2020; Valentinova et al., 2023). We delineated the neural circuit mechanisms within these structures that underlie pain-related aversion and fear (Figure 2).

### 5.1 ACC

The ACC critically encodes the unpleasantness of chronic pain and its related negative affect. This function is underpinned by pathological neuronal hyperexcitability, driven by a dysregulation of excitatory/inhibitory balance involving glutamatergic hyperactivity, GABAergic impairment, and astrocytic activation (Guan et al., 2025; Hu et al., 2025; Meda et al., 2019; Valentinova et al., 2023; Wang et al., 2024). Chronic pain conditions result in substantial plasticity within the S1 → ACC circuit, marked by increased functional connectivity and subsequent hyperexcitability of ACC neurons, which intensifies the processing of pain-related aversion (Singh et al., 2020). At the cellular level, nerve injury (SNI) diminishes the activity of vasoactive intestinal polypeptide interneurons in the PL, leading to the disinhibition of PL glutamatergic projections to the ACC and the subsequent development of pain-related aversion (Li M. et al., 2022).

Additionally, inputs from the BLA selectively enhance the excitability of layer II/III pyramidal neurons in the ACC through targeted projections, thereby facilitating pain-induced aversive responses and fear avoidance behaviors (Valentinova et al., 2023). Research on thalamic modulation indicates that inputs from the mediodorsal thalamic nucleus (MD) to the ACC intensify pain-related aversion by selectively inhibiting layer V subcortical projection neurons, thereby disrupting the local excitation/inhibition balance within these neuronal populations (Meda et al., 2019). Importantly, hyperactive layer V pyramidal neurons in the ACC project excitatory signals to both dopamine D2 receptor-expressing medium spiny neurons in the nucleus accumbens (NAc) and GABAergic neurons in the VTA, establishing a distinct neural pathway that mediates aversive behaviors associated with neuropathic pain (Gao et al., 2020).

Collectively, these findings underscore the ACC as a critical convergence point for multiple pain-related circuits, where the integration of sensory and affective information occurs through layer-specific neuronal interactions and long-range projections to limbic and reward systems.

### 5.2 Thalamus

The study found that the anterior part of the paraventricular thalamic nucleus (PVA) exhibited VgluT2 neuronal excitability under the condition of chronic inflammatory pain, which was directly related to the mechanical pain hypersensitivity and aversive behaviors of the chronic pain mice. Although recent studies have suggested that the bed nucleus of the stria terminalis (BNST) is involved in the generation and persistence of pain-related anxiety-like behaviors (Fang et al., 2025). However, the research conducted by Mindaye et al. (2024) revealed that the PVA → BNST pathway mediates pain-like hypersensitivity, but is not related to the emotional expression of chronic pain. The activation of the PVA-NAc pathway is sufficient to trigger aversion, but does not lead to mechanical hypersensitivity. The different subgroups of PVA glutamatergic neurons have distinct projections and mediate the aversion caused by pain and mechanical hypersensitivity. This understanding may lead to more targeted pain management strategies.

### 5.3 Amygdala

Exposure to pain states triggers associative fear memories (Stegemann et al., 2023). The CeL plays a key role in regulating both the acquisition and expression of fear. The research found that nerve injury may impair fear extinction through neuronal hyperexcitation in the CeLC (Ji et al., 2018). CeL-on (fired by conditioned stimuli and partially overlapping with SOM neurons), CeL-off (suppressed by conditioned stimuli and partially overlapping with PKC-δ neurons), and CeM neurons, mediate fear conditioning (Hevesi et al., 2021; Whittle et al., 2021). Inhibition of CeM neurons by CeL-off neurons reduces freezing behavior. Silencing CeL-off neurons and secretagogin neurons in CeL (a Ca^2+^ sensor protein and a subset of PKC-δ neurons) results in the disinhibition of CeM neurons, which increases freezing behavior (Hevesi et al., 2021; Whittle et al., 2021). These same neuronal populations aforementioned are also important in pain perception (Li and Sheets, 2020; Wilson et al., 2019), suggesting that the fear-regulating microcircuit in the CeA may similarly modulate pain-related fear, though direct evidence remains limited.

The CeA receives projections from multiple brain regions. The lateral amygdala (LA) and BLA are the main inputs to the CeA, integrating multimodal sensory inputs from both cortical and thalamic regions and subsequently processing this information to encode emotional-affective valence before transmitting it to the CeA (Thompson and Neugebauer, 2017). Reduced excitability of the BLA → CeL glutamatergic projection loop induces post-traumatic stress disorder-related anxiety and fear (Gao et al., 2024). The paraventricular thalamus (PVT) neurons target CeL and preferentially innervate CeL^SOM^ neurons, which are involved in the expression of conditioned fear and the storage of fear memory (Penzo et al., 2015). The posterior PVT (pPVT) promotes the stability of fear memory and fear expression by promoting the excitability and synaptic plasticity of CeL^SOM^ neurons through brain derived neurotrophic factor (BDNF)/tropomysin-related kinase B (Penzo et al., 2015). In particular, the connection of pPVT and CeA is also involved in the regulation of pain (Liang H. Y. et al., 2020) mediates pain-induced fear response remains ambiguous. The neuropathic pain is linked to strengthened projection from ventral hippocampus (vHPC) to CeA, which leads to the activation of CeA^GABA^ neurons, and prompts the chronicity of pain and the expression of fear memory (Kami et al., 2022). Pain-induced fear and anxiety are associated with elevated FosB^+^ expression in CA1 neurons, which have projections to the BLA. Reversing the activation of the vHPC → amygdala circuit are the routs through which voluntary running can promote the extinction of contextual fear memory (Minami et al., 2023).

The CeA serves as a convergence hub for pain and fear processing, integrating inputs from the BLA, PVT, and vHPC to regulate fear memory formation, persistence, and extinction. While distinct neuronal subpopulations (SOM, PKC-δ, secretagogin) modulate both pain and fear, further research is needed to clarify whether pain-specific fear responses rely on the same circuitry.

## 6 Pain-related anxiety and depression

**Figure 3:**
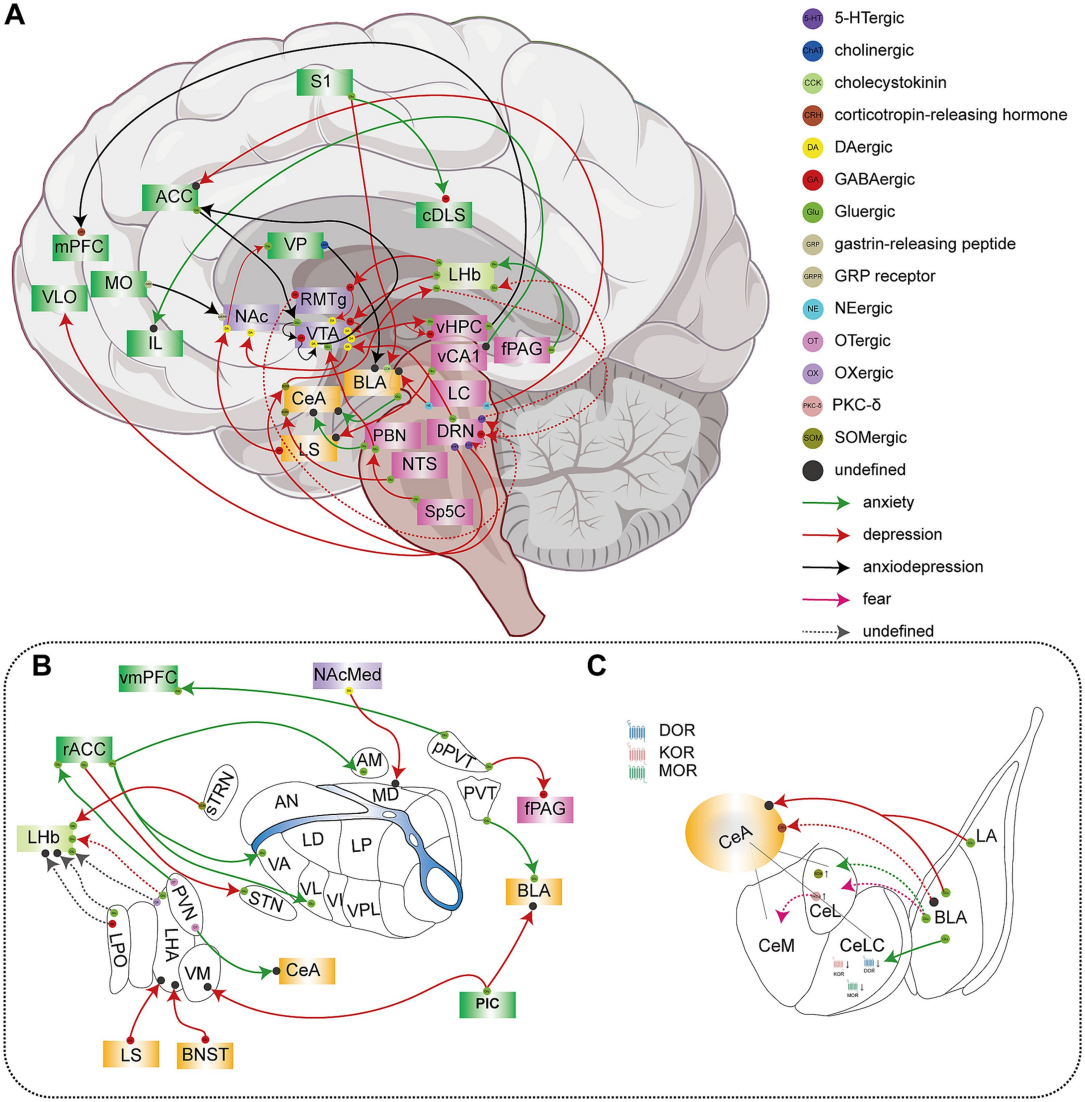
A schematic depiction of neural circuits associated with pain-related behaviors, including anxiety and depression. Solid lines represent established neural circuits, while dashed lines indicate neural circuits that may be involved in pain comorbid with depression or anxiety. Green arrows denote neural circuits involved in the regulation of chronic pain-related anxiety-like behaviors: S1 → cDLS, rACC → VA/VL/AM, vCA1 → IL, fPAG → LHb, BLA → CeA, BLA → CeLC, PBN → CeA, PVT → BLA, pPVT → vmPFC, PVN → CeA. Red arrows denote neural circuits involved in the regulation of chronic pain-related depression-like behaviors: S1 → BLA, LHb → RMTg → VTA, LHb → VTA, DRN → CeA → LHb, DRN → VTA → NAc, Sp5C → PBN → VTA, NTS → CeA, LC → BLA, LC → ACC, sTRN → LHb, rACC → STN, LS → LHA, BNST → LHA, PIC → VM/BLA, LA/BLA → CeA. Black arrows represent neural circuits involved in the regulation of both chronic pain-related anxiety- and depression-like behaviors: vHPC → mPFC, VP → BLA, ACC → VTA. **(A)** A schematic coronal brain section with key anatomical regions delineated and color-coded. **(B)** Depiction of thalamus-associated neural circuits implicated in the comorbidity of chronic pain with anxiety and/or depression. **(C)** Detailed view highlighting specific intra-amygdala connections and regional interactions.

Chronic pain frequently elicits adverse emotional responses, including anxiety and depression. This article provides a comprehensive review of the neural circuit mechanisms underlying pain-related anxiety and depression, highlighting the involvement of various brain nuclei, including the ACC, mPFC, amygdala, LHb, VTA, PVT, and LHA (Figure 3).

### 6.1 ACC

Emerging evidence from neural circuit studies reveals that the ACC orchestrates pain-associated negative emotional states via distinct thalamocortical and midbrain circuits (Shen et al., 2020; Song et al., 2024; Wang et al., 2024; Xue et al., 2022). glutamatergic efferent circuits from the rostral anterior cingulate cortex (rACC) to various thalamic nuclei, including the anterior medial thalamus, ventral anterior thalamic nucleus, and ventrolateral thalamic nucleus, have been identified as neural substrates that underlie anxiety-like behaviors associated with pain (Shen et al., 2020). Similarly, glutamatergic neuronal projections from the ACC to the subthalamic nucleus initiate pain-related depression-like behaviors (Wang et al., 2024). The activation of oxytocinergic neurons originating in the hypothalamic paraventricular nucleus (PVN) and projecting to the ACC can alleviate both neuropathic pain and associated anxiety-like behaviors (Li et al., 2021). The positive feedback loop of ACC^Glu^ → VTA^GABA^ → VTA^DA^ → ACC^Glu^ mediates the duration and severity of pain as well as pain-related anxiodepressive-like behaviors (Song et al., 2024). Furthermore, the LC → ACC circuit mediates chronic pain and pain-induced depression-like behaviors, whereas bilateral chemogenetic inhibition or blockade of the α-adrenoceptor activity within the rACC can alleviate pain-induced depression (Llorca-Torralba et al., 2022).

### 6.2 PFC

The mPFC responds to harmful stimuli and is the central hub for mental comorbidities associated with chronic pain (Condés-Lara et al., 1989; Kummer et al., 2020). Electrophysiological studies in SNI models reveal two key pathological features: (i) significantly enhanced theta oscillations in the mPFC, and (ii) increased theta-band synchronization between mPFC and vHPC, both strongly correlating with anxiety-like behavioral manifestations (Sang et al., 2018; Usdin and Dimitrov, 2016). Fortunately, administration of serotonin reuptake inhibitors or intra-mPFC injections of serotonin (5-HT) is effective in relieving neuropathic pain-associated anxiety-like behaviors (Sang et al., 2018). A recent article reported that trigeminal pain activates glutamatergic neural projections from vHPC to mPFC, targeting the activation of the CRH receptor 1 (CRHR1) signaling pathway in mPFC (Lv et al., 2024). This led to feedforward inhibition of mPFC layer 5 pyramidal neurons and fostering anxiodepressive-like behaviors in trigeminal pain animal models. CRHR1 or γ-aminobutyric acid receptor subtype A (GABA_A_R) antagonists alleviate pain-induced anxiodepression-like behaviors. Additionally, BDNF deficiency has been shown to sit at the core of ventral hippocampal CA1 (vCA1) → IL circuit disruption in CFA-induced spontaneous pain rats, whereas activation of the vCA1 → IL circuit or overexpression of BDNF can alleviate pain and anxiety symptoms (Ma et al., 2019).

The ventrolateral orbitofrontal cortex (VLO) and medial orbitofrontal cortex (MO) are significant sub-region of the prefrontal cortex, integral to the integration of emotions, motivations, and decision-making processes. Empirical studies have demonstrated that the VLO and MO is essential in mediating neuropathic pain in rodent models, as well as the associated anxiety and depressive behaviors (Zhao T. et al., 2025; Zhao Y. et al., 2025). At the neural circuit level, research indicates that activation of the serotoninergic (5-HTergic) projections from the dorsal raphe nucleus (DRN) to the VLO exerts an antidepressant effect. This effect can be inhibited by the 5-HT2A antagonist MDL100907 or the 5-HT1A antagonist WAY100635. The underlying mechanism involves the activation of the DRN → VLO pathway, which stimulates the release of 5-HT, directly exciting glutamatergic neurons within the VLO via 5-HT2A receptors and activating GABAergic interneurons through 5-HT1A receptors. This process alleviates the inhibition of glutamatergic neurons. The synergistic activation of 5-HT1A and 5-HT2A receptors in the VLO, along with the enhancement of the VLO → DRN circuit activity, can mitigate depressive-like behaviors induced by neuropathic pain (Sheng et al., 2025). This discovery offers valuable insights for developing therapeutic strategies for depression associated with neuropathic conditions. Research has demonstrated that the gastrin-releasing peptide (GRP)/GRP receptor (GRPR) system within the MO to NAc pathway plays a pivotal role in modulating chronic pain and its associated emotional aspects. Specifically, chronic pain has been shown to decrease the excitability of NAc^GRPR^ neurons and the release of GRP from the MO to the NAc. Dysfunction within the MO^GRP^ → NAc^GRPR^ pathway is implicated in the development of heterodynia, anxiety-like behaviors, and aversive responses associated with chronic pain. These findings are relevant to both neuropathic (SNI) and non-neuropathic (CFA) chronic pain models (Zhao T. et al., 2025).

### 6.3 Amygdala

Gabaergic neurons in CeA regulate pain-related negative emotions. The research found neural network connections between nucleus of the solitary tract (NTS) and CeA are involved in the regulation of pain-induced depression-like behaviors (He et al., 2022). NTS^Glu^ → CeA^SOM^ is a particular neural circuit that mediates pain-related depression-like behavior, but is not involved in chronic stress-related depression. BLA is the major input nucleus of the amygdala, which relays injury information to CeA and regulates the sensory as well as affective components of pain (Jiang et al., 2021; Usdin and Dimitrov, 2016). At 35 days after spinal nerve ligation (SNL), SNL rats show a substantial decrease in sucrose preference and prolonged immobility in the forced swimming test (Jiang et al., 2021). This may be related to the enhanced long-term synaptic depression (LTD) of LA/BLA → CeA synapses generated by GluA2-containing α-amino-3-hydroxy-5-methyl-4-isoxazolepropionic acid (AMPA) receptors endocytosis. Zhou et al. (2021) suggested both DOR1 and DOR2 become dysfunctional when acute pain turns into chronic pain, and anxiety-like behaviors emerge. The CeLC receives multimodal input from BLA. The use of opioids to activate μ-opioid receptor (MOR), some DOR, and KOR in CeA can reduce the transmission of BLA → CeLC nociceptive signals and reduce the output of synaptic injury signals, which is beneficial to reduce pain and the negative emotions induced by pain. Endogenous opioid receptors can regulate the link between BLA and CeA, and some opioid receptor subtypes are characterized by a time-dependent mechanism. Therefore, the role of each subtype in pain and related negative emotionsmust be accurately grasped.

CRF-expressing neurons in CeA are widely distributed throughout pain- and emotion-processing networks. Optogenetic activation of CeA^CRF^ neurons in sham animals recapitulates key features of chronic pain states, including hyperalgesia and anxiety-like behaviors (Mazzitelli et al., 2022). Ji and his coworkers (Ji et al., 2017) discovered that the knockdown of the 5-HT receptor isoform (5-HT_2C_R) in BLA inhibits pathological neuropathic pain and associated emotional affective responses such as anxiety and depression. CRF1 and 5-HT_2C_R are interlinked and the knockdown of 5-HT_2C_R can abolish the effects of 5-HT_2C_R agonists and CRF1 receptor antagonists, whereas CRF1 receptor antagonists prevent the effects of 5-HT_2C_R agonists (Ji and Neugebauer, 2019). In brief, the BLA → CeA circuit, targeting the 5-HT_2C_R-CRF1 signaling pathway, mediates pain and related negative emotions (such as anxiety and depression).

A previous study revealed that CCI long-term (5–6 weeks) rats have a reduced pain threshold as well as exhibit anxiety-like behaviors, cognitive deficits, and significant freezing behavior (Llorca-Torralba et al., 2019). These are closely related to the hyperactivation of the LC → BLA circuit, and the elevated activity of β-adrenergic receptors (β-ARs) brought on by chronic pain. β-ARs are neural substrates of anxiety-like behaviors induced by light stimulation, and they can be activated by LC^NE^ neurons. Inhibiting LC → BLA projection or reducing β-ARs activity can cure pain-induced anxiety. BLA^Glu^ neuron hyperactivity is essential for processing pain’s sensory and emotional aspects (Meng et al., 2022). BLA receives glutamatergic inputs from MD/ insular cortex (IC) (Meng et al., 2022), posterior insular cortex (pIC) (Chen et al., 2024) and cholinergic (ChAT) projections from the ventral pallidal (VP) (Ji et al., 2023). All of this activates the excitability of BLA neurons. The MD/IC → BLA circuit regulates the sensory and affective components of pain (Meng et al., 2022). The pIC → BLA/ventromedial nucleus (VM) circuit and regulates the pain and related depression-like behaviors (Chen et al., 2024). Activation of the VP^ChAT^ → BLA circuit contributes to hyperalgesia and pain-related anxiodepressive-like behaviors (Ji et al., 2023). Furthermore, Delta-containing GABA_A_R at the synapses of BLA neurons project to vHPC and the anterodorsal bed nucleus of the stria terminalis. Lifting the inhibition of the former and strengthening the inhibition of the latter is an important mechanism through which anxiety is induced following chronic social failure and stress (Qin et al., 2022). This mechanism may represent a common pathway for both stress-induced and pain-related anxiety.

### 6.4 LHb

The LHb serves as a critical neural hub that integrates and modulates multiple neurophysiological processes, including nociception, affective states (anxiety and depression), and cognitive functions (Hu et al., 2020). Evidence indicates that LHb-mediated regulation of these processes depends on neuronal hyperexcitability, burst-firing activity, and synaptic plasticity (Cui et al., 2020; Hu et al., 2020).

Research has demonstrated that the hyperexcitability of LHb^Glu^ neurons plays a significant role in the development of trigeminal neuralgia and related anxiety-like behaviors (Cui et al., 2020; Zhang et al., 2023). Zhang et al. (2023) have clarified that glutamatergic neurokinin B (NKB)-positive neurons located within front section of the periaqueductal gray (fPAG) project to the LHb, specifically interacting with tachykinin receptor 3 (NK3R), which is encoded by the *Tacr3* gene. The chemogenetic suppression of NKB release in the fPAG negated the beneficial effects of *Tacr3* overexpression on allodynia and anxiety-like behaviors induced by partial transection of the infraorbital nerve (pT-ION). The fPAG^NKB^ → LHb^Glu^ pathway regulates orofacial allodynia and anxiety behaviors in pT-ION mice by modulating Tacr3 and NK3R activities within the LHb.

Furthermore, neurons in LHb also receive inputs from lateral preoptic area of the hypothalamus Gulergic (LPO^Glu^) neurons, which significantly upregulate MOR mRNA expression (Waung et al., 2022). Administration of DAMGO (a MOR-selective agonist) within LHb is beneficial in reversing the sensory and affective experience of pain in rats, possibly by activating MOR, which inhibits the monosynaptic inputs to LHb from LPO^Glu^ neurons. A different study suggested that foot shock stimulation promotes the development of mental illnesses like depression by upsetting the glutamatergic or GABAergic balance of LPO inputs to LHb (Barker et al., 2017). This suggests that LPO^Glu^ → LHb projections critically regulate pain and affective states. Notably, the LPO → LHb circuit may offer a novel target for opioid analgesia without the rewarding effects of traditional opioids (Waung et al., 2022). The LHA is a subnuclei of the thalamus. Neuropathic pain activates the glutamatergic neurons in LHA (LHA^Glu^), contributing to depression and memory and learning impairments associated with chronic pain (Gu et al., 2023; Meng et al., 2024). Inhibition of LHA^Glu^ neuronal activity or inhibition of the LHA → LHb circuit facilitates relief of neuropathic pain (Gu et al., 2023). Under restraint stress, LHA transmits stress information to the LHb through glutamatergic neurons, which promote the generation of depression-like behaviors (Zheng et al., 2022). The LHA → LHb circuit may modify the excitability of LHb neurons, leading to pain-related negative emotions, especially depression.

Zhou et al. (2019) discovered that chronic pain reliefs the inhibition of 5-HT neurons in DRN (DRN^5-HT^) and local SOM^−^ or SOM^+^ neurons in CeA, resulting in enhanced CeA^SOM+^ neuron activity and direct projection to LHb^Glu^ neurons, which jointly mediated the comorbidity of chronic pain and depression. Injection of 5-HT1A receptor agonists into CeA could reverse the disinhibition of CeA^SOM^ neurons and relieve pain-induced depression-like behaviors. In addition, DRN has 5-HTergic projections to the LHb, and the 5-HT1b receptor may mediate the inhibition of LHb by the DRN (Zhang et al., 2018). Optogenetic activation of the DRN → LHb circuit in chronic unpredictable mild stress rats decreases immobility time during the forced swimming test and increases the desire for sucrose (Zhang et al., 2018). As a result, there might be a fascinating neurological closed loop between LHb and DRN, which could represent a crucial component of the comorbidity of pain and depression.

Wang et al. (2023b) discovered that neurons in the sensory thalamic reticular nucleus (sTRN) that release somatostatin (SOM) send inhibitory projections to LHb. Under conditions of chronic pain or stress, this inhibitory circuit is compromised, characterized by suppressed synaptic strength. This loss of inhibition leads to hyperexcitability in the LHb, thereby promoting depressive-like behaviors. Enhancing activation of the sTRN^SOM^ → LHb^Glu^ circuit relieves depression-like behaviors generated by chronic pain chronic stress and chronic pain; thus, suggesting that this circuit could be a viable target for eliminating pain-induced depression. According to Wang et al. (2021) activating the projection of orexinergic neurons in LHA (LHA^OX^) to LHb^Glu^ can reverse depression or anxiety-like behaviors induced by chronic social stress. Activating OX in LHA may have analgesic and antidepressant benefits (Wang et al., 2021; Wang C. et al., 2022). OX might work through the LHA^OX^ → LHb^Glu^ circuit and may be a potential target for the pain and related affective disorder therapy. The LHA → LHb circuit may modify the excitability of LHb neurons, leading to pain-related negative emotions, especially depression.

### 6.5 VTA

VTA is primarily composed of DAergic neurons involved in the control of reward-related behaviors (Flores-García et al., 2023). As a critical confluence of pain and depression, VTA receives projections from LHb. Based on a study (Zhang C. et al., 2024), the glutamatergic neuron projection of LHb to VTA inhibits VTA^GABA^ neurons, which further inhibits VTA^DA^ neurons and promotes the susceptibility to depression-like behaviors in mice with chronic pain. Particularly, through RMTg, LHb can indirectly regulate VTA neuronal activity, and this LHb → RMTg → VTA pathway is also implicated in the regulation of pain-related depression (Liu Y. et al., 2025; Taylor et al., 2019; Xin et al., 2022). In addition, activation of the DRN^Glu^ → VTA^DA^ circuit is beneficial to the release of DA neurons in the NAc medial shell, which alleviates pain and anhedonia via D2 and D1 receptors, respectively (Wang et al., 2023a). Furthermore, as noted earlier, activation of VTA^DA^ neurons by LPBN glutamatergic inputs induces pain-related depression (Zhang et al., 2021). Together, these findings establish that the VTA acts as a pivotal integrator, translating diverse aversive and protective signals into motivated behavior. Multiple distinct glutamatergic pathways—from the LHb, LPBN, and DRN—all ultimately converge to modulate the activity of VTA^DA^ neurons highlights a shared neural substrate for pain-affect comorbidity. Therefore, therapeutic strategies that suppress aversive inputs (e.g., from LHb or LPBN) or enhance protective ones (e.g., from DRN) may rebalance VTA output and restore motivational homeostasis, offering novel circuit-based targets for alleviating the affective symptoms of chronic pain.

Evidence further delineates a specialized medial VTA DAergic projection to the vHPC that is anatomically distinct from other mesolimbic pathways (Ji et al., 2025). This circuit is selectively suppressed in depression-susceptibility, and its activation reverses depressive-like behaviors without affecting pain, indicating a specific role in affective control. The antidepressant effect requires co-activation of vHPC D1 receptors on pyramidal neurons and D2 receptors on GABAergic interneurons. In contrast, the classical VTA^DA^ → NAc reward circuit modulates both sensory and affective components of pain (Yang et al., 2023). Furthermore, evidence establishes VTA → NAc glutamatergic inputs as a critical pathway mediating chronic pain-induced anxiety and depression (Abdul et al., 2022). Collectively, these findings illustrate a functional dissociation within VTA efferent pathways, revealing an additional layer of complexity in VTA-mediated affective regulation.

### 6.6 NAc

Recent research has elucidated that projections from the vCA1 region to NAc and the thalamic reticular nucleus (TRN) are selectively involved in the regulation of nociceptive hypersensitivity responses. Conversely, projections targeting the lateral septum (LS) are specifically implicated in the modulation of depressive-like behaviors (Meng et al., 2026). A study identified a reduction in the activity of medium spiny neurons (MSNs) expressing D1 and D2 dopamine receptors within the medial nucleus of the accumbens (NAcMed) in conditions of chronic pain accompanied by comorbid depression. Activation of D1-MSNs was found to mitigate depressive-like behaviors, whereas activation of D2-MSNs yielded analgesic effects (Xia et al., 2025). Notably, The research conducted by Liu D. et al. (2025) indicates that NAcMed^D2-MSNs^ neurons mediate neuropathic pain and depressive-like behaviors. This may be attributed to the different projection circuits of NAcMed D2 neurons. Current evidence indicates that the AM^Glu^ → NAcMed^D2-MSNs^ → LHA^OX^ neural circuit is involved in mediating pain-related behaviors in SNI mice, without influencing depressive-like symptoms. Conversely, the LS^GABA^ → NAcMed^D2-MSNs^ → VP^Glu^ neural circuit modulates chronic pain-related depressive-like behaviors, but does not affect depressive-like behaviors that are not associated with pain (Liu D. et al., 2025). Furthermore, the NAcMed^D1-MSN^ → MD pathway appears to specifically regulate pain-related depressive behaviors (Xia et al., 2025). These findings suggest that D1-MSNs and D2-MSNs in the NAcMed differentially regulate the emotional and sensory dimensions of chronic pain comorbid with depression through separate neural circuits, highlighting potential circuit-specific targets for precise therapeutic interventions addressing this comorbidity.

### 6.7 PVT

PVT is a midline thalamus nucleus that regulates sleep–wake states, pain, and emotional responses such as reward, anxiety, depression, and stress (Bu et al., 2022). Under neuropathic pain conditions, glutamatergic neurons within the PVT exhibit heightened activity and transmit excitatory projections to the BLA (Tang et al., 2024). The application of AMPA or N-methyl-D-aspartate (NMDA) receptor antagonists to the BLA has been found to decrease both heat and mechanical pain associated with the activation of the PVT → BLA, while concurrently alleviating anxiety-like behaviors related to pain (Tang et al., 2024). In addition, research (Liang H. Y. et al., 2020; Liang S. et al., 2020) suggests that pain signals from the pPVT can trigger the release of glutamate, which can activate neuronal nitric oxide synthase (nNOS) in ventromedial prefrontal cortex (vmPFC). This enhances nNOS activity and increases nitric oxide (NO) production, leading to increased transport of AMPA receptor within vmPFC vertebral neurons. Specifically, NO diffusion in vmPFC favors S-nitrosylation of stargazin (an AMPA receptor-interacting protein) and N-ethylmaleimide-sensitive factor, which may facilitate enhanced AMPA receptor trafficking and mediate pain-related anxiety-like behaviors (Chen et al., 2023). The research found that, the neural circuit involving pPVT^Glu^ to GABAergic neurons in the ventrolateral periaqueductal gray (vlPAG^GABA^) was implicated in the manifestation of chronic pain behaviors, while the pathway from PVA^Glu^ to NAc D1 to D2 was associated with the depression-like behaviors induced by SNI (Deng et al., 2024). The interaction between these two pathways facilitates the development of comorbidities following nerve injury, potentially offering valuable insights for devising optimal treatments for the comorbidity of chronic pain and depression.

### 6.8 PVN

The PVN and supraoptic nucleus as well as microcellular and paracellular neurons located in the accessory nucleus are responsible for producing oxytocin (OT) (Wang P. et al., 2022). OT produces anxiolytic, antidepressant, and analgesic effects through complex neurological mechanisms that are closely related to PVN^GABA^ neuron activity, GABAergic transmission in the brain, and noradrenergic signaling in the SC (Iwasaki et al., 2023; Wang C. et al., 2022). A recent study (Li et al., 2023) showed that anxiety-like behaviors produced by inflammatory pain reduce the activity of PVN^OT^ neurons and that the activation of the PVN^OT^ → CeA projection can significantly decrease pain-induced anxiety. Nasal feeding OT is beneficial to restore down-regulated BDNF in the dentate gyrus and ultimately alleviate the comorbidity of neuropathic pain and depression (Liu R. et al., 2024; Liu Y. et al., 2024; Zeng et al., 2022). Enhancing the projection of OT from the hypothalamus to the hippocampus may be a potential treatment for neuropathic pain. Further, the projection of OT to NAc, insula (IS), ventral striatum (VS), and PFC is involved in the regulation of pain, and may also affect the emotional component of pain (Herpertz et al., 2019; Liu et al., 2023). Among them, OT appears to regulate the cognitive and fear responses to pain via IS and VS, respectively (Herpertz et al., 2019). However, the sources of these OT remain to be further studied.

### 6.9 LHA

The LHA is responsible for mediating responses to pain and controlling the negative emotions associated with chronic pain (Meng et al., 2024). Using retrograde tracing, GABAergic neurons in the LS are shown to project to LHA and that activation of this pathway induces the comorbidity of pain and anxiety (Wang D. et al., 2023). This study also demonstrated that neurons in LS that project widely to multiple brain areas but solely to LHA mediate pain and anxiety comorbidity. Additionally, the GABA neurons in BNST that send messages to LHA also produce pain and anxiety (Yamauchi et al., 2022). And recent studies have found that the BNST is conducive to the formation of persistent anxiety induced by pain (Fang et al., 2025). The GABAergic input to LHA may be one of the neurobiological mechanisms of pain-related anxiety. Notably, the precise targets of these GABAergic neurons within the LHA remain to be elucidated.

## 7 Descending modulation of pain

**Figure 4:**
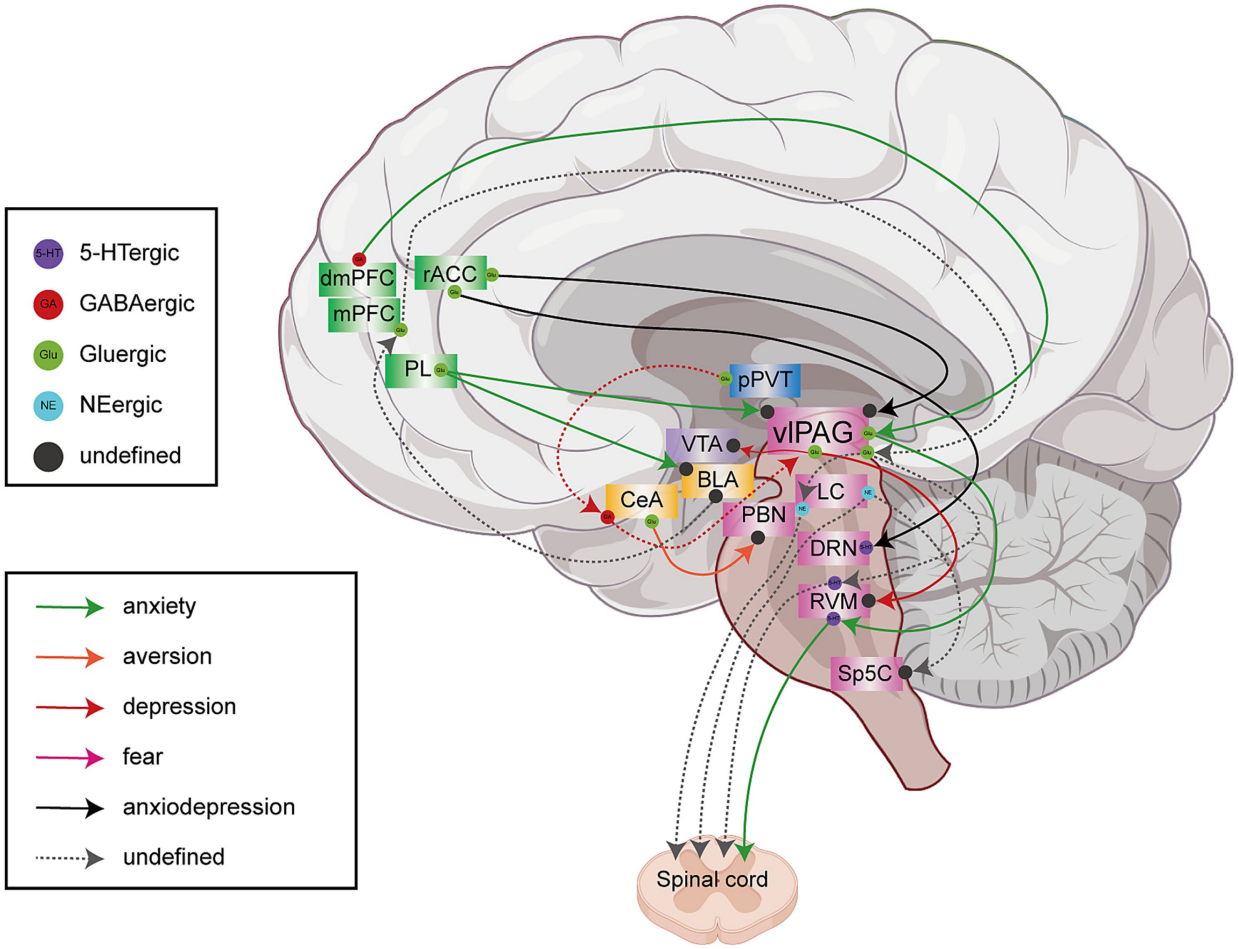
Schematic diagram of descending neural circuits underlying abnormal emotions and cognitive impairments associated with chronic pain. This schematic illustrates the descending neural circuits involved in mediating abnormal emotions and cognitive impairments associated with chronic pain. The depicted circuits include pathways such as amygdala → cortex → PAG, thalamus → amygdala → PAG, CeA → PBN, and LC → SC/Sp5C, among others. Specifically, the rACC → vLPAG and rACC → DRN neural circuits co-regulate anxiety and depressive emotions comorbid with chronic pain. The dmPFC → vlPAG → RVM → SC and PL → vlPAG/BLA circuits are implicated in anxiety symptoms comorbid with chronic pain. The vlPAG → VTA/RVM pathway participates in the regulation of depression-related emotions associated with chronic pain, while the CeA → PBN circuit is involved in mediating pain-related aversion.

The descending pain modulatory system is a neural mechanism by which the brain exerts bidirectional control over pain perception through descending pathways. This system primarily modulates nociceptive inputs and spinal neuronal activity to either inhibit or facilitate pain. It comprises functionally distinct yet interconnected neural circuits, with key structures including midbrain and brainstem nuclei such as the periaqueductal gray (PAG) and the rostral ventromedial medulla (RVM) (Bannister et al., 2025). Increasing evidence suggests that dysfunction within these descending pain pathways contributes to the chronicity of pain and the emergence of associated negative emotions (Figure 4).

### 7.1 PAG

The PAG is a key center for integrating ascending and descending pain pathways, processing multisensory nociceptive signals. It is vital for modulating various physiological and pathological activities, including the perception of pain, defensive and aggressive actions, anxiety, and depression (Behbehani, 1995; Zhang H. et al., 2024). The PAG combines inputs from cortical and subcortical areas to control different behaviors (Bagley and Ingram, 2020; Bandler and Keay, 1996).

#### 7.1.1 Amygdala → cortex → PAG

Huang et al. (2019) discovered that the dysfunction of the BLA → mPFC → vlPAG → SC circuit may be the key to hyperalgesia and abnormal emotions in neuropathic pain. The abnormal synaptic transmission caused by endocannabinoid depletion, as well as the decreased excitability of vlPAG to input from LC and the increased inhibition of vlPAG to input from RVM, results in decreased serotonin and norepinephrine input to SC, which is closely related to the dysfunction of this circuit. The overexcitation of the dorsomedial prefrontal cortex (dmPFC) inhibitory neurons disrupts the normal transmission of the dmPFC^Glu^ → vlPAG circuit, which promotes the development of pain and the generation of anxiety (Yin et al., 2020). Furthermore, abnormal dmPFC^Glu^ → vlPAG signaling also affects the projection of glutamatergic neurons from vlPAG to RVM, potentially influencing pain-induced anxiety-like behaviors. Alternatively, inhibiting metabotropic glutamate receptor 1 and GABA_A_R or activating the dmPFC^Glu^ → vlPAG circuit produces analgesic and anxiolytic effects (Yin et al., 2020).

According to Gao et al. (2023) neurons in PL that project to BLA (PL^BLA^) and neurons that project to the vlPAG (PL^l/vlPAG^) are distinct and modulate separately emotional and sensory dimensions of pain. In SNI mice, the firing frequency of PL^l/vlPAG^ neurons decreases while the frequency of spontaneous inhibitory postsynaptic currents increases and both the firing frequency and EPSC amplitude of PL^BLA^ neurons increase. Tumor necrosis factor-α (TNF-α) and its receptor (TNFR1) are upregulated in SNI mice, which leads to the enhancement of postsynaptic GluA1 receptor expression, increased excitatory synaptic activity of PL^BLA^ neurons, and induction of anxiety-like behaviors though not hyperalgesia. Inhibition of the PL → vlPAG circuit and activation of the PL → BLA circuit promote pain and related anxiety-like behaviors, respectively.

Further research elucidates that the descending pain modulatory pathway plays a pivotal role in mediating the analgesic and anxiolytic effects of electroacupuncture (Xu et al., 2023; Zhu et al., 2021). Empirical evidence demonstrates that activation of the glutamatergic rACC → vlPAG circuit provokes pain and anxiety-like behaviors in Sham mice while concurrently inhibiting electroacupuncture-induced analgesia (Zhu et al., 2021). In contrast, electroacupuncture mitigates pain and anxiety in SNI mice through the activation of the rACC → DRN glutamatergic neural circuit (Xu et al., 2023). These findings provide a neurobiological foundation for the dual therapeutic actions of electroacupuncture.

#### 7.1.2 Thalamus → amygdala → PAG

The study discovered that pPVT glutamatergic neurons are activated by chronic pain (Liang S. et al., 2020) and nociceptive hypersensitivity and anxiety brought on by nerve damage can be reduced by selectively inhibiting PVT glutamatergic neurons (Tang et al., 2024). Using the SNI neuropathic pain model and retrograde synaptic tracing based on rabies virus and cell type specificity, Liang S. et al. (2020) discovered that the circuit consisting of pPVT^Glu^ → CeA plays a critical role in regulating pain. The vlPAG^Gul^ neurons are the downstream targets of the pPVT → CeA circuit, whose excitation facilitates the development of chronic pain and associated depression-like behaviors (Lee et al., 2023; Liang S. et al., 2020). Furthermore, vlPAG projects to RVM to regulate pain, projects to VTA to regulate emotion, and damage to the vlPAG^Gul^ neurons is conducive to pain sensitivity and depression-like behaviors (Lee et al., 2023). Nevertheless, investigating how the pPVT → CeA → vlPAG circuit regulates the projection of vlPAG to its downstream targets remains an interesting topic as it may help us understand the process underlying the development of pain-related negative emotions. However, the extent to which it regulates pain-related negative emotions requires further investigation.

### 7.2 CeA → PBN

Besides conveying upward nociceptive signals, the PBN plays a role in modulating descending circuits involved in the emotional processing of pain (Raver et al., 2020). Studies have found that LPBN accepts CeA projections and this circuit is a novel target for alleviating pain and associated negative emotions (Hogri et al., 2022; Raver et al., 2020). The CeA^CAM^ neurons, a subset of CeA neurons that express the *CaMKIIα* gene, project to LPBN neurons, which express SOM, CRH, dynorphin, and other factors to produce inhibitory neuronal effects (Hogri et al., 2022; Raver et al., 2020). The activation of this circuit can induce analgesia and produce positive emotional responses, which is beneficial for promoting the rehabilitation of patients with chronic pain and comorbid emotional disorders.

### 7.3 LC → SC/Sp5C

As a key relay, the LC is integral to the descending pain modulation system (Kong et al., 2023). The output of LC to the SC is a component of the descending analgesic circuit, which contrasts with the ascending pathway action of LC (Hirschberg et al., 2017; Llorca-Torralba et al., 2022; Usdin and Dimitrov, 2016). Chemogenetic activation of neurons in LC that project to SC raises the withdrawal threshold and causes conditioned place preference in neuropathic pain animal models (Hirschberg et al., 2017). According to a recent study (Li J. et al., 2022), selective activation of the LC → SC circuit induces more NE release, which may directly affect the NE receptor α2β-AR in microglia and ultimately inhibit its activation and lessen neuroinflammation. In diabetic mice with orofacial pain, enhancing the activity of α2-NE receptors in the spinal dorsal horn leads to more LC^NE^ neurons projecting to Sp5C, which may increase the inhibition of Sp5C by LC, thereby alleviating chronic orofacial pain (Mesa-Lombardo et al., 2023). Activation of the LC → SC/Sp5C neural circuits is an effective way to relieve pain. However, further studies are required to investigate whether the LC → SC and LC → Sp5C circuits mediated by NEergic neurons influence the affective dimension of pain.

## 8 Summary and prospects

Through a systematic review of the neural circuitry underlying chronic pain and its comorbidities, we find that the associated emotional and cognitive dysfunctions arise not from generalized brain impairment but from specific disruptions within distinct neural circuits (Table 1). These circuits, while performing unique roles, are interconnected through key hub regions in the brain, creating a positive feedback loop with the descending pain modulation system. The overlapping brain regions, serving as pivotal hub areas, have the capacity to connect various behavioral outcomes associated with chronic pain (e.g., ACC, amygdala). This understanding directly associates clinical symptoms with specific neuroanatomical substrates, providing a robust foundation for elucidating the mechanisms underlying comorbidities and for developing circuit-based biomarkers and targeted therapies (Table 2). Future treatment strategies should transition from focusing solely on “analgesia” to emphasizing “circuit repair,” by selectively targeting the neural pathways that correspond to a patient’s predominant symptoms.

**Table 1:** The “↑” arrows indicate the activation of neural circuits.

Neural circuits	Type of pain	Pain model	Function (pathology)	The sensations aspect of chronic pain	The emotional aspect of chronic pain	References
LC^NE^ → PFC	NPP	SNL	↓	Chronic pain	Cognitive function	Suto et al. (2014)
BLA → CeA	NPP	SNL	↑	Chronic pain	Anxiety and depression	Ji et al. (2017)
mPFC^Glu^ → HPC	NPP	SNI	↑	Chronic pain	Cognitive function	Cardoso-Cruz et al. (2019)
MD^Glu^-ACC	NPP	Taxol/SNI	↑	—	Aversive	Meda et al. (2019)
vCA1 → IL	IFP	CFA	↓	Chronic pain	Anxiety	Ma et al. (2019)
LC^EN^ → BLA	NPP	CCI	↑	Chronic pain	Anxiety	Llorca-Torralba et al. (2019)
DRN^5-HT^ → CeA^SOM^ → LHb^Glu^	NPP	SNI mice	↓	—	Depression	Zhou et al. (2019)
S1^Glu^ → ACC	NPP and IFP	SNI/CFA	↑	—	Aversion	Singh et al. (2020)
S^1Glu^ → cDLS^GABA^	IFP	CFA		Chronic pain	Anxiety	Jin et al. (2020)
ACC^Glu^-NAc^DA^/VTA^GABA^	NPP	CCI	↑	—	Aversive	Gao et al. (2020)
rACC^Glu^-VA/VL/AM	NPP	CFA	↑	—	Anxiety	Shen et al. (2020)
pPVT^Glu^ → vmPFC	IFP	CFA mice	↑	Chronic pain	Anxiety	Liang H. Y. et al. (2020)
dmPFC^Glu^ → vlPAG	NPP	CPNL mice	↓	Pain	Anxiety	Yin et al. (2020)
LA/BLA^Glu^ → CeA	NPP	SNL	↑	Chronic pain	Depression and aversive	Jiang et al. (2021)
PBN^Glu^ → CeA	IFP	CFA	↑	Chronic pain	Anxiety	Zhou et al. (2021)
PVN^OT^ → ACC	NPP	CPNL	↓	Chronic pain	Anxiety	Li et al. (2021)
Sp5C^Glu^ → LPBN^Glu^ → VTA^DA^	TN	pIONT	↑	—	Depression	Zhang et al. (2021)
rACC^Glu^-vlPAG	NPP	SNI	↑	Pain	Anxiety	Zhu et al. (2021)
vHPC^Glu^-CeA^GABA^	NPP and IFP	—	↑	Chronic pain	Fear	Kami et al. (2022)
PL^Glu^-ACC^GABA^	NPP	SNI	↓	Chronic pain	Cognitive function	Li M. et al. (2022)
LC^EN^ → rACC	NPP	CCI	↓	—	Depression	Llorca-Torralba et al. (2022)
NTS^Glu^ → CeA^SOM^	NPP	Oxaliplatin	↑	—	Depression	He et al. (2022)
IC^Glu^ → BLA	IFP	CFA	↑	Chronic pain	Aversive	Meng et al. (2022)
MD^Glu^ → BLA	IFP	CFA	↑	—	Aversive	Meng et al. (2022)
LPO^Glu^ → LHb	NPP	SNI rat	↑	Chronic pain	Aversive	Waung et al. (2022)
VTA^Glu^ → NAc	NPP and IFP	CCI/CFA	↑	Chronic pain	Anxiety	Abdul et al. (2022)
BNST^GABA^-LHA	NPP	SNI	↑	—	Anxiety	Yamauchi et al. (2022)
LPBN^PACAP^ → CeA	Chronic pain	—	↑	Chronic pain	Anxiety	Seiglie et al. (2023)
BLA^Glu^ → ACC	NPP	SNI	↑	—	Aversive and fear	Valentinova et al. (2023)
LHb^Glu^ → VTA^GABA^ → VTA^DA^	IFP	CFA	↑	Chronic pain	Cognitive function	Alemi et al. (2023)
BLA^Glu^ → ACC	NPP and IFP	SNI/CFA	↑	—	Cognitive functions	Valentinova et al. (2023)
VP^ChAT^-BLA	NPP and IFP	SNI/Capsaicin	↑	Chronic pain	Anxiety and depression	Ji et al. (2023)
fPAG^Glu^ → LHb^Glu^	TN	pT-ION mice	↑	Chronic pain	Anxiety	Zhang et al. (2023)
sTRN^SOM^ → LHb^Glu^	NPP	SNI	↑	—	Depression	Wang et al. (2023b)
DRN^Glu^ → VTA^DA-^NAcMed^D2R/D1R^	NPP	SNI	↓	Chronic pain	Anhedonia	Wang et al. (2023a)
VTA^DA^ → NAc	IFP	CFA	↓	Chronic pain	Depression and anxiety	Yang et al. (2023)
PVN^OT^ → CeA	IFP	CFA mice	↓		Anxiety	Li et al. (2023)
LS^GABA^-LHA	IFP	CFA	↑	Chronic pain	Anxiety	Wang D. et al. (2023)
rACC^Glu^-DRN	NPP	SNI	↓		—	Xu et al. (2023)
vlPAG^Gul^-RVM	NPP	SNL	↓	Chronic pain	—	Lee et al. (2023)
vlPAG^Gul^-VTA	NPP	SNL	↓	—	Depression	Lee et al. (2023)
PL-BLA/vlPAG PL^Glu^ → BLA	NPP	SNI mice	↑	—	Anxiety	Gao et al. (2023)
PL^Glu^ → vlPAG	NPP	SNI mice	↓	Pain	—	Gao et al. (2023)
PVA^Glu^-BNST	IFP	Formalin	↑	Chronic pain	—	Mindaye et al. (2024)
PVA^Glu^-NAc	IFP	Formalin	↑	—	Aversive	Mindaye et al. (2024)
ACC^Glu^ → VTA^GABA^ → VTA^DA^ → ACC^Glu^	NPP	SNI	↑	Chronic pain	Anxiety	Song et al. (2024)
vHPC^Glu^ → mPFC^CRH^	TN	CION	↑	—	Anxiety and depression	Lv et al. (2024)
pIC^Glu^ → BLA/VM	NPP	SNI	↑	Chronic pain	Depression	Chen et al. (2024)
LHb^Glu^ → VTA^GABA^ → VTA^DA^	NPP	SNI	↑	Chronic pain	Depression	Zhang C. et al. (2024)
PVT^Glu^ → BLA^Glu^	NPP	CCI mice	↑	Chronic pain	Anxiety	Tang et al. (2024)
pPVT^Glu^ → vlPAG^GABA^	NPP	SNI	↑	—	Depression	Deng et al. (2024)
PVA^Glu^ → NAc D1 → D2	NPP	SNI	↓	Chronic pain	—	Deng et al. (2024)
S1HL^Glu^ → BLA^CCK^	NPP	SNI	↑	—	Depression	Chen et al. (2025)
LH^bGlu^ → RMTg^GABA^ → VTA^DA^	NPP	SNI	↑	Chronic pain	Cognitive function and depression	Liu Y. et al. (2025)
DRN^5-HT^-VLO	TN	CION	↓	—	Depression	Sheng et al. (2025)
MO^GRP^ → NAc^GRPR^	NPP and IFP	SNI/CFA	↓	Chronic pain	Anxiety and depression	Zhao T. et al. (2025)
VTA^DA^-vHPC	NPP	SNI	↓	Chronic pain	Depression	Ji et al. (2025)
AM^Glu^ → NAcMed^D2-MSNs^ → LHA^OX^	NPP	CFA	↓	Chronic pain	—	Liu D. et al. (2025)
LS^GABA^ → NAcMed^D2-MSNs^ → VP^Glu^	NPP	CFA	↓	—	Depression	Liu D. et al. (2025)
NAcMed^D1-MSN^ → MD	NPP	CFA	↓	—	Depression	Xia et al. (2025)
vCA1^Glu^-NAc vCA1^Glu^-TRN	NPP	CCI mice	↓	Chronic pain	—	Meng et al. (2026)
vCA1^Glu^-LS	NPP	CCI mice	↓	—	Depression	Meng et al. (2026)

“↓” arrows indicate inhibition of neural circuits. “—” indicates no change in the target function. CCI, chronic contractile injury; CFA, complete Freund’s adjuvant; CION, chronic constriction injury of the infraorbital nerve; CPNL, common peroneal nerve ligation; IFP, inflammatory pain; NPP, neuropathic pain; pIONT, partial infraorbital nerve transection; pT-ION, partial transection of the infraorbital nerve; SNI, spared nerve injury; SNL, spinal nerve ligation; TN, trigeminal neuralgia.

**Table 2:** The “↑” indicate upregulation of target function or symptomatic improvement.

Targets	Neural circuits	Location	Model	Function (pathology)	Intervention	Mechanism	Effect	References
5-HT_2C_R	BLA → CeA	BLA	SNL rat	↑	Knockdown 5-HT_2C_R	Block the increased excitatory transmission from BLA to CeA	Pain↓ Anxiety↓ Depression↓	Ji et al. (2017)
5-HT_1A_Rs	DRN^5-HT^ → CeA^SOM^ → LHb^Glu^	CeA postsynaptic	SNI mice	*	Selective 5-HT1A R agonist	Reverse the disinhibition of CeA^SOM^ neurons	Depression↓	Zhou et al. (2019)
β-ARs	LC^EN^ → BLA	BLA	CCI rat	↑	Nonselective β-AR antagonist propranolol	*	Pain↓ Anxiety↓	Llorca-Torralba et al. (2019)
BDNF	vCA1 → IL	vCA1 → IL	CFA rat	↓	Overexpression	Reverses electrophysiological changes	Pain↓ Anxiety↓	Ma et al. (2019)
MOR	BLA^Glu^ → CeLC and PBN^Glu^ → CeLC	BLA → CeLC and PBN → CeLC synapses	Rat	—	MOR agonists	Glutamatergic release from BLA and PBN to CeLC↓	Pain↓ negative emotions↓	Kissiwaa et al. (2020)
GABA_A_R mGluR1	dmPFC^Glu^ → vlPAG	dmPFC	CPNL mice	↑	GABA_A_R or mGluR1 antagonist	*	Pain↓ Anxiety↓	Yin et al. (2020)
AMPAR	pPVT^Glu^ → vmPFC	vmPFC	CFA mice	AMPAR trafficking and function↑	Exogenous stargazin mutant/nNOS-PSD-95 inhibitors	S-nitrosylation↓ NO production↓	Pain↓ Anxiety↓	Chen et al. (2023) and Liang H. Y. et al. (2020)
OT/OTR	PVN^OT^ → ACC	OT in PVN OTR in ACC	CPNL mice	OT↑ OTR↑	Intra‐ACC inject oxytocin /activate the PVN^OT^ → ACC circuit	ACC pre-LTP ↓ Ratio of excitatory/inhibitory transmission in the ACC↓	Pain↓ Anxiety↓	Li et al. (2021)
DOR2	PBN^Glu^ → CeA	PBN presynaptic	CFA mice	Disappearance	DOR2 agonists	*	Pain↓ Anxiety↓	Zhou et al. (2021)
GluA2	LA/BLA^Glu^ → CeA	LA/BLA → CeA postsynaptic	SNL rat	LTD at the LA/BLA-CeA synapse↑	pep2-EVKI or Tat-GluA2(3Y)	Disruption of GluA2-containing AMPAR endocytosis and trafficking	Pain↓ Depression↓ Aversive states↓	Jiang et al. (2021)
DA	Sp5C^Glu^ → LPBN^Glu^ → VTA^DA^	VTA	pIONT mice	↑	Inhibit the VTA ^DA^ neurons (retigabine)	*	Depression↓	Zhang et al. (2021)
MOR	LPO^Glu^ → LHb	LHb	SNI rat	—	MOR agonists	Inhibits the LPO^Glu^ → LHB circuit	Pain↓ and related Aversive↓	Waung et al. (2022)
α-adrenoreceptor (α1/α2)	LC^EN^ → rACC	rACC	CCI rat	↑	α2/α1-adrenoreceptor antagonist (prazosin/idazoxan)	*	Depression↓	Llorca-Torralba et al. (2022)
AMPAR	BLA^Glu^ → ACC	BLA → ACC synapses	SNI mice	AMPAR transmission↑	Low-frequency stimulation BLA → ACC	Restores AMPAR function	Cognitive functions↑	Valentinova et al. (2023)
PACAP	LPBN → CeA	LPBN	CCI rat	↑	PACAP receptor (PAC1) antagonist	CeA ERK signal transduction↓	Pain↓ Anxiety↓	Seiglie et al. (2023)
NK3R	fPAG^Glu^ → LHb^Glu^	LHb	pT-ION mice	↓	Senktide (a selective NK3R agonist)	Reversal of the hyperexcitation of LHb^Glu^ neurons	Pain↓ Anxiety↓	Zhang et al. (2023)
TNF-α/TNFR1 GluA1	PL^Glu^ → BLA	PL	SNI mice	↑	TNF-α antagonist/knockdown TNFR1/blockade of postsynaptic GluA1 insertion into PL^BLA^ neurons	sEPSC↓ The activity of PL^BLA^ neurons↓	Anxiety↓	Gao et al. (2023)
OT/OTR	PVN^OT^ → CeA	OT in PVN, OTR in CeA	CFA mice	OT↓	Intra‐CeA inject oxytocin/activate the PVN^OT^-CeA circuit	*	Anxiety↓	Li et al. (2023)
D2R D1R	DRN^Glu^ → VTA^DA-^NAcMed^D2R/D1R^	NAcMed	SNI mice	*	Eticlopride/SCH23390	—	Chronic pain and anhedonia	Wang et al. (2023a)
NMDAR AMPAR	PVT^Glu^ → BLA^Glu^	BLA	CCI mice	Regulate the excitatory glutamatergic synaptic connections of BLA → PVT	AMPA or NMDA receptor antagonists	Inhibit the PVT → BLA neural circuit	Pain↓ Anxiety↓	Tang et al. (2024)
CRHR1 GABA_A_R	vHPC^Glu^ → mPFC^CRH^	vHPC^Glu^ → mPFC^CRH^/mPFC Postsynaptic	CION mice	↑	CRHR1 or GABA_A_R antagonist	Block CRH and GABA signaling	Anxiety↓ Depression↓	Lv et al. (2024)
DR2	ACC^Glu^ → VTA^GABA^ → VTA^DA^ → ACC^Glu^	Postsynaptic D2R in ACCGlu neurons/presynaptic D2R in VTADA neurons	SNI mice	↓	Quinpirole in ACC/haloperidol in VTA	mimics VTA^DA^–ACC transmission/restore the decreased activity of VTADA neurons	Pain and anxiety	Song et al. (2024)
5-HT1A and 5-HT2A receptors	DRN5-HT-VLO	VLO	CION mice	↓	DOI in VOL/8-OH-DPAT in VLO	Increases the activity of the DRN-VLO pathway	Depression↓	Sheng et al. (2025)
CRP	MO^GRP^ → NAc^GRPR^	MO NAc	SNI mice CFA mice	↓	GRP supplementation in NAc/activate MO^GRP^ neurons projecting to the NAc	Increase the content of CRP in the NAc region	Pain↓ Anxiety↓ Aversive↓	Zhao T. et al. (2025)

“↓” arrows indicate downregulation of target function or symptom relief. “—” indicates no change in the target function. “*” indicates that the function and action mechanism are unclear. CCI, chronic contractile injury; CFA, complete Freund’s adjuvant; CION, chronic constriction injury of the infraorbital nerve; CPNL, common peroneal nerve ligation; pIONT, partial infraorbital nerve transection; pT-ION, partial transection of the infraorbital nerve; SNI, spared nerve injury; SNL, spinal nerve ligation.

For example, deep brain stimulation exhibits therapeutic potential by precisely modulating synaptic connections within specific neural circuits, such as the BLA → CeL → anterodorsal bed nucleus of the stria terminalis, to mitigate anxiety (Gao et al., 2024). This review builds upon the understanding of circuit mechanisms to summarize potential circuit-based therapeutic targets implicated in pain and related maladaptive behaviors. Neuroimaging techniques, including functional magnetic resonance imaging and electroencephalography, further aid in diagnosis and treatment planning by identifying alterations in functional connectivity (Vanneste and De Ridder, 2021; Zhu et al., 2024). Nonetheless, a significant challenge persists in the absence of non-invasive tools that can monitor and modulate specific cell types within deep brain circuits in humans over extended periods, with adequate spatiotemporal resolution. Furthermore, the translation of circuit-level discoveries into systemically deliverable and brain-region-specific pharmaceuticals remains a formidable challenge.

Future research should focus on the following key directions: verifying whether abnormal activation in chronic pain-related brain circuits occurs within critical time windows; exploring whether activity patterns of different circuits can predict specific comorbidity types (e.g., anxiety-dominant or depression-dominant); developing imaging- or electrophysiology-based diagnostic methods, as well as non-invasive diagnostic and therapeutic techniques such as transcranial magnetic stimulation; and clarifying whether abnormalities in related brain regions act as pathogenic “drivers” or compensatory responses to guide precise treatment. These directions will advance the diagnosis and treatment of pain comorbidities toward precision medicine.

## Glossary

5-HT_2C_R: 5-HT receptor isoform
5-HTergic: Serotonergic
ACC: Anterior cingulate cortex
AMPA: α-Amino-3-hydroxy-5-methyl-4-isoxazolepropionic acid
AN: Anterior nucleus
BDNF: Brain derived neurotrophic factor
BLA: Basolateral amygdala
BMA: Basomedial amygdala
BNST: Bed nucleus of the stria terminalis
CaMKIIα: Calcium/calmodulin-dependent protein kinase-II α
CCI: Chronic contractile injury
CCK: Cholecystokinin
cDLS: Caudal dorsolateral striatum
CeA: Central amygdala
CeL: Lateral CeA
CeLC: Laterocapsular region
CeM: Medial CeA
CFA: Complete Freund’s adjuvant
ChAT: Cholinergic
CION: Chronic constriction injury of the infraorbital nerve
CPNL: Common peroneal nerve ligation
CRF: Corticotropin-releasing factor
CRH: Corticotropin-releasing hormone
CRHR1: CRH receptor 1
DAergic: Dopaminergic
dmPFC: Dorsomedial prefrontal cortex
DOR1: Delta opioid receptor 1
DOR2: Delta opioid receptor 2
DRN: Dorsal raphe nucleus
EPSC: Excitatory postsynaptic current
fPAG: Front section of the periaqueductal gray
GABA_A_R: γ-aminobutyric acid receptor subtype A
GABAergic: Gamma-aminobutyric acid
GRP: Gastrin-releasing peptide
GRPR: GRP receptor
HPC: Ventral hippocampus
IC: Insular cortex
IL: Infralimbic medial prefrontal cortex
IPSC: Polysynaptic inhibitory postsynaptic current
IS: Insula
KOR: Kappa opioid receptors
LA: Lateral amygdala
LC: Locus coeruleus
LHA: Lateral hypothalamic area
LHb: Lateral habenular
LP: Lateral posterior nucleus
LPBN: Lateral parabrachial nucleus
LPO: Lateral hypothalamic preoptic area
LS: Lateral septum
LTD: Long-term synaptic depression
LTP: Long-term potentiation
MD: Mediodorsal thalamus
mGluR1: Metabotropic glutamate receptor 1
MO: Medial orbitofrontal cortex
MOR: μ-opioid receptor
mPFC: Medial prefrontal cortex
MSNs: Medium spiny neurons
NAc: Nucleus accumbens
NAcMed: Medial nucleus of the accumbens
NEergic: Norepinephrine-ergic
NK3R: Tachykinin receptor 3
NKB: Neurokinin B
NMDA: N-methyl-D-aspartate
nNOS: Neuronal nitric oxide synthase
NO: Nitric oxide
NTS: Nucleus of the solitary tract
OT: Oxytocin
PACAP: Pituitary adenylate cyclase-activating polypeptide
PAG: Periaqueductal gray
PBN: Parabrachial nucleus
PFC: Prefrontal cortex
pIC: Posterior insular cortex
pIONT: Partial infraorbital nerve transection
PKC-δ: Protein kinase C-delta
PL: Prelimbic medial prefrontal cortex
pPVT: Posterior paraventricular thalamic nucleus
pT-ION: Partial transection of the infraorbital nerve
PVA: Anterior part of the PVT
PVN: Paraventricular nucleus of hypothalamus
PVT: Paraventricular thalamic nucleus
rACC: Rostral anterior cingulate cortex
RMTg: Rostromedial tegmental nucleus
RVM: Rostral ventromedial medulla
S1: Primary somatosensory cortex
SC: Spinal cord
sEPSC: Spontaneous excitatory postsynaptic current
SNI: Spared nerve injury
SNL: Spinal nerve ligation
SOMergic: Somatostatinergic
Sp5C: Spinal trigeminal subnucleus caudalis
SSC: Somatosensory cortex
STN: Subthalamic nucleus
sTRN: Sensory thalamic reticular nucleus
*Tacr1*: *Tachykinin receptor 1*
TNFR1: TNF-α receptor
TNF-α: Tumor necrosis factor-α
TRN: Thalamic reticular nucleus
VA: Ventral anterior thalamus
vCA1: Ventral hippocampal CA1
vHPC: Ventral hippocampus
VI: Ventral intermediat nucleus
VL: Ventrolateral thalamus
VLO: Ventrolateral orbitofrontal cortex
vlPAG: Ventrolateral periaqueductal gray
VM: Ventromedial nucleus
vmPFC: Ventromedial prefrontal cortex
VP: Ventral pallidum
VPL: Ventral posterolateral nucleus
VTA: Ventral tegmental area
β-ARs: β-adrenergic receptors
